# Testing the Power of Antiseptics

**Published:** 1886-06

**Authors:** W. D. Miller

**Affiliations:** Berlin, Germany


### TESTING THE POWER OF ANTISEPTICS.

BY DR. W. D. MILLER, BERLIN, GERMANY.

From time to time experiments, purporting to determine the disinfecting power of specified substances, appear in the dental journals, which, so far from accomplishing the object desired, show so great a want of knowledge of the ordinary methods used in determining the strength of antiseptics, that a few words on the subject may not be out of place.

A writer in a recent number of this journal, for instance, concludes that a given substance has no disinfecting power because it produced no chemical change upon sulphuretted hydrogen.

A disinfectant, according to the definitions of Koch, Fluegge and others, is a substance which retards the development of micro-organisms, and in proper concentration kills them; for most purposes the more inert the substance is chemically, the better; in fact, the chemical action of many antiseptics is one of the greatest impediments to their free use in medicine. Even for disinfecting rags, clothing, sick-rooms, etc., we do not call for chemical changes which would be absolutely harmful, but simply for destruction of germs.

A very simple method of determining the strength of any antiseptic, as well as its rapidity of action, is the following, which will also be found substantially in previous numbers of the INDEPENDENT PRACTITIONER:

(1.) To determine the lowest degree of concentration necessary to prevent the development of a given fungus, prepare a large number of tubes, each containing, say, 5,0 cc. of a sterilized nutrient solution (say beef-extract 1,5, peptone 1,5, sugar 1, water 100). Add to the first tube a sufficient quantity of the substance to make its strength 1 to 10. To the second tube add enough to give a strength of 1 to 20, then 1 to 50, 1 to 100, 1 to 1000, etc. Infect these solutions with the fungus to be tested, and then keep them at a temperature favorable to its development. In all tubes which remain clear, the concentration is sufficiently strong to prevent the development of the fungi; in those which become cloudy, not. By the first experiment we establish the fact that the limit lies between certain numbers, say 1 to 4000 and 1 to 5000. Repeating the experiment with 1 to 4000 as highest and 1 to 5000 as lowest concentration, we find the limit somewhere between 1 to 4500 and 1 to 4600; a third experiment will then determine at just what concentration development ceases.

To determine the rapidity with which a certain substance (in solution) acts, proceed as follows: Place a few drops of the substance to be tested in a sterilized watch-glass; add to this, on a loop of platinum wire, a minute drop of a bacterium culture, stirring it quickly with the wire; then transfer, at given intervals, a very small drop to culture tubes, which keep at a favorable temperature for the development of the fungi. If the exposure was sufficiently long to devitalize the fungi the tubes will remain clear; otherwise they will become cloudy in a few hours, or in two or three days at most.

See also INDEPENDENT PRACTITIONER, 1885, page 412.

